# Bridging the gap between healthcare professions’ regulation and practice: the “lived experience” of community pharmacists in Ireland following regulatory change

**DOI:** 10.1186/s40545-022-00465-5

**Published:** 2022-10-29

**Authors:** Matthew Lynch, Naonori Kodate, Conor Hickey, Aisling C. O’Leary

**Affiliations:** 1 School of Pharmacy and Biomolecular Sciences, University of Medicine and Health Sciences, Royal College of Surgeons in Ireland, 1st Floor Ardilaun House Block B, 111 St. Stephen’s Green, Dublin 2, DO2 VN51 Ireland; 2 School of Social Policy, Social Work and Social Justice, University College Dublin, Dublin, Ireland; 3 Public Policy Research Center, Hokkaido University, Sapporo, Japan; 4 L’École des Hautes Études en Sciences Sociales, Paris, France; 5 Institute for Future Initiatives, University of Tokyo, Tokyo, Japan; 6 Universal Accessibility and Ageing Research Centre, Nishitokyo, Japan; 7 National Centre for Pharmacoeconomics, St. James’s Hospital, Dublin 8, Ireland

**Keywords:** Healthcare Professions regulation, Implementation, Lived experience, Pharmacist, Regulation, Responsive regulation

## Abstract

**Background:**

Reforms to models of health and care regulation internationally have adapted to address the challenges associated with regulating healthcare professionals. Pharmacists in Ireland entered a new era of regulation with the enactment of the Pharmacy Act in 2007 which significantly updated the law regulating pharmacy in Ireland and expanded the regulatory scope considerably. An earlier study in 2017 examined the experiences of 20 community pharmacists of the Act. This follow-up study aimed to expand the scope of the original study to all community pharmacists in Ireland, to report their “lived experience” of the regulatory model introduced by the Act, assessing its impact on their professional practice using the principles of “better regulation”.

**Methods:**

Survey methodology was used to assess the perception of all community pharmacists registered with the Pharmaceutical Society of Ireland of the Act, as implemented, on their practice using an experimental design based on the seven principles of “Better Regulation”. Descriptive statistics analyzed quantitative responses while answers from open-ended questions were analyzed using a combination of a modified framework analysis and a qualitative content analysis.

**Results:**

Respondents agreed that the Act was necessary, although its implementation by the regulator was largely not viewed as fulfilling the remaining “Better Regulation” principles of being effective, proportional, consistent, agile, accountable and transparent. In particular, its proportionality was questioned. This resulted in pharmacists perceiving that their professional competency to act in the best interests of their patients was not appropriately acknowledged by the regulator, which in turn compromised their ability to provide optimal care for their patients.

**Conclusion:**

While healthcare professional regulation must primarily be concerned with public protection, it must also have regard to its impact on those delivering healthcare services. The findings highlight the challenge internationally of balancing rigidity and flexibility in professional health and care regulation, and the importance of a regulatory conversation occurring between those regulating and those regulated. This would serve to promote mutual learning and understanding to create a responsive approach to regulation, underpinned by mutual trust, effective risk assessment and adherence to the principles of “Better Regulation”.

## Background

Regulation is a key component of contemporary governance, with a proliferation of regulatory agencies, extensive separation of policy-making from operational tasks and the formalization of previously informal relationships [1–3]. Regulation of healthcare professionals globally is an integral part of wider healthcare governance within the context of the welfare state [4–7]. Countries continuously seek to improve the quality of their healthcare systems to make them safer for patients [8, 9]. People expect an optimal outcome when they avail of a healthcare service, but inevitably, situations arise whereby patients experience an adverse health outcome, which is seldom attributable to one specific cause. For the patient concerned, an error in their care may result in life-altering or life-ending consequences. If the rationale underlying the regulation of healthcare is for patient protection and safety, then the model of regulation in place should minimize the likelihood of harm arising for any patient.

The recent global pandemic has reinforced the view that universal access to public health and care systems are essential to the wellbeing of a nation, and pharmacists across the globe have been an integral part of the healthcare systems, playing a vital role in patient care and support as well as infection control [10]. The contributions made by community pharmacists in various parts of the world to the prevention, preparedness and response to the COVID-19 crisis have been reported [11–14]. Yet there has been relatively little research conducted on regulation and its impact on pharmacists, with a few exceptions [15–21].

Regulation of health professionals including pharmacists has traditionally been viewed as a matter of voluntary compliance by the individual, supported by professional self-regulation [1, 22]. As the high cost of harm to patients, healthcare systems and societies began to be recognized in the late 1990s, regulation has been undergoing significant reform in many post-industrial economies [23, 24]. This was characterized by a shift away from the traditional model of pure self-regulation by professionals themselves to one of *“regulated (self-) regulation”*, which is subject to greater external scrutiny and audit [25]. The model of ‘responsive regulation’ advocates regulators being responsive to the conduct of those they regulate when determining what regulatory action, if any, is required [26]. It requires specialized skills and knowledge, and therefore carries many challenges, including how to balance rigidity and flexibility [27, 28]. The incorporation of trust in the model of regulation is important as lack of trust tends to produce overregulation [29, 30]. Accordingly, in healthcare, regulatory bodies increasingly should learn from their interactions with the regulated and adjust their practices [4]. In health professional self-regulation, regulatory bodies are usually established by statute to discharge these functions on behalf of the State, in the public interest. These healthcare regulators are mandated with protecting those in receipt of services and with supporting standards within the professions that they regulate [31].

The model of healthcare professional regulation in Ireland including pharmacy is essentially one of mandated self-regulation. Professional groups are subject to statutory regulation but are generally free as a professional grouping to implement and regulate in accordance with the State’s statutory framework. A new Health & Social Care Professionals Act was introduced in 2005 to formally regulate various allied healthcare professionals [32]. The Medical Practitioners and Pharmacy Acts were enacted in 2007 to update the regulation of doctors and pharmacists and pharmacies respectively, while in 2011, the Nurses and Midwives Act updated the regulation of nurses and midwives [33–35]. Regulation of dentists in Ireland continues in accordance with the Dentists Act introduced in 1985 [36].

The introduction of the Pharmacy Act in 2007 significantly updated the law regulating pharmacy in Ireland [34]. It replaced previous Acts dating from 1875 to 1962 and expanded the regulatory scope considerably, particularly in the area of discipline and continuing professional development (CPD). Prior to the 2007 Act, there was no procedure in place to investigate complaints about registered pharmacists or the operation of retail pharmacy businesses (RPBs) also known as community pharmacies. The Pharmaceutical Society of Ireland (PSI) is the regulatory body charged under the Act with implementing its provisions. It has extensive powers to investigate and adjudicate upon complaints about pharmacists and pharmacies which, if upheld, may result in the cancellation of their registration.

The Pharmacy Act 2007 mandated a number of changes to disciplinary procedures for pharmacists, inspection and enforcement by the regulator and the education and training of pharmacists. These changes are generally in line with those in other English-speaking countries with legal systems based on common law [17]. In relation to inspection, the PSI introduced the Pharmacy Assessment System (PAS) in 2017, a self-audit system for community pharmacists (CPs) to assess their compliance with various parameters to complement and support the formal inspection process. Regarding education, the PSI introduced a requirement for higher educational institutions to introduce a 5-year integrated program of studies for pharmacy students wishing to register as a pharmacist [37]. As part of this integrated Master of Pharmacy (MPharm) program, the traditional single 12-month period of supervised practical training that was undertaken following completion of the four year program, is now divided into two blocks with 4 months completed in Year 4 and 8 months in Year 5. It is a function of the PSI to ensure that pharmacists undertake CPD.

Notwithstanding a commitment by the Irish government to keep its regulatory institutions and frameworks under review, it has not undertaken any assessment of the regulatory impact of Acts regulating healthcare professionals [38]. The Organization for Economic Co-operation and Development (OECD), recommends that regulators should conduct periodic reviews of regulations following their implementation and that the legislature should monitor and periodically review that the system of regulation is working as intended [39]. For the system of regulation to be responsive, having the voice of those regulated heard is critical. The importance of expanding the “*regulatory conversation*” beyond the expert regulators to include those who are the subject of regulation and their “*lived experience*” of regulation is recognized [40, 41]. Both in Ireland and elsewhere, the dearth of published literature on the assessment of the impact of regulation on health professionals, may be due to the fact that study of the regulation of health professionals does not have a robust and well-defined identity as an academic discipline or field of study [42]. Methodological challenges in the conduct of such research are also a contributing factor [43].

Lynch and Kodate first addressed this lacuna in the literature with their review of the Pharmacy Act and its implementation in the 10-year period following its introduction [44]. They used a theoretical framework developed from the perspective of both the theory of implementation and the principles of regulation aligned with the model of responsive regulation [26, 45–47]. Seven regulatory principles were identified which acted as the framework for the conduct of the study and its subsequent data analysis, collectively known as the principles of *“Better Regulation”* (Table 1) [38, 39, 48].

**Table 1:** Study principles of “Better Regulation’’ adapted from [38, 49]

Necessity	Is the regulation necessary, reduce red tape, still valid?
Effectiveness/targeted	Is the regulation properly targeted, properly complied with and enforced?
Proportionality	Do the advantages of the regulation outweigh its disadvantages?
Transparency	Are stakeholders consulted prior to regulating? Is the regulation clear and accessible to all?
Accountability	Is the regulation clear as to precisely who is responsible to whom and for what? Is there an effective appeals process?
Consistency	Does the regulation give rise to anomalies and inconsistencies? Is best practice developed in one area applied when regulating other areas?
Agility	Is the regulation capable of adapting to anticipate change?

The Lynch and Kodate study was the first of its kind in Ireland and it reported that CPs acknowledged the need for regulation but perceived that the PSI needed to adopt a more responsive approach to implementation, if the Act is to be considered a model of better regulation. It highlighted the importance of regulation having the capacity to strike an appropriate balance between rules and the practitioner’s professional judgement, while continuing to ensure adequate accountability [44]. However, it was a small qualitative study among 20 CPs which may not have been truly representative of the wider CP population.

To address this potential lack of generalizability, a larger survey was undertaken using the same theoretical framework. The aim was to establish how CPs (the regulatees) have experienced the model of regulation introduced by the 2007 Pharmacy Act as implemented by the PSI and to determine their understanding of its alignment with, and fulfillment of, the principles of better regulation.

## Methods

An online self-administered survey was used to address the research aim. The survey consisted of both closed and open-ended questions in three parts, where Part 1 consisted of demographic details of the respondents, Part 2 encompassed questions relating to the “*lived experience*” of CPs with the new Pharmacy Act and Part 3 assessed CPs’ understanding of the seven principles of “Better Regulation” as relating to the Act’s implementation.

The survey was piloted among 5 CPs and refined prior to wider dissemination.

All CPs as notified to the PSI, totaling 3732, were invited to participate in the study and the survey was distributed through the online SurveyMonkey^®^ tool.

Quantitative data were collated in Microsoft Excel^®^, and descriptive statistics were used to describe respondents’ demographic details. Responses to individual questions were reported as proportions of categorical variables. Chi-squared tests of independence were performed in R to examine if hypothesized associations between respondent characteristics and their responses to survey questions/statements were statistically significant at a significance level of 5%. Qualitative data from open-ended questions were analyzed using a combination of a modified framework analysis based on the seven principles of better regulation and a qualitative content analysis [50–54]. In order to ensure impartiality and reflexivity, the research team, comprising skilled researchers in both qualitative and quantitative study design from a range of relevant disciplines (pharmacy, social science and statistics), engaged in a systematic process to review the responses and reach consensus on the thematic categories to be extracted in the data [55]. Approval for the study was obtained from the Research Ethics Committee at the Royal College of Surgeons in Ireland (REC. No. 201908007).

## Results

A total of 308 CPs responded to the survey, while 228 completed the full survey (response rate 6.1%). The majority of respondents were female (54%), aged between 30 and 60 years of age (84%), while 40% were first registered as a pharmacist outside of Ireland. The respondents were categorized into: (i) those who had practiced under the preceding Pharmacy Acts (72%, *n* = 165) and those who had not (28%, *n* = 63); (ii) owners of RPBs (31%, *n* = 71) and employee pharmacists (69%, *n* = 157); (iii) management grade (60%, *n* = 137) and non-management grade (49%, *n* = 91).

### (a) Attitudes to components of Pharmacy Act

#### Disciplinary procedures

Almost one half of respondents (47%, *n* = 107) felt that the disciplinary procedures were not implemented in a manner that appropriately balanced the need to protect the public availing of pharmacy services with upholding the rights of a CP to fair procedures (Table 2). Respondents who had practiced under the preceding Pharmacy Acts, RPB owners and management grade pharmacists were more likely to hold this view. Most respondents (51%, *n* = 69) indicated that they did not know if the provision to use mediation to resolve complaints was appropriately utilized by the PSI.

**Table 2:** Attitudes of CPs to components of Pharmacy Act

Community pharmacist position	Response			*p*-value from Pearson’s Chi-squared test
	Yes	No	Don’t know	
Appropriate disciplinary procedures				
Owner of RPB	9 (13%)	47 (66%)	15 (21%)	Owners of RPBs more likely to disagree, *p*<0.001
Employee (permanent)	26 (22%)	44 (37%)	48 (41%)	
Employee (relief)	17 (44%)	16 (41%)	6 (15%)	
Total	52 (23%)	107 (47%)	69 (30%)	
Use of mediation appropriate				
Owner of RPB^#^	5 (7%)	37 (52%)	29 (41%)	Owners of RPBs more likely to disagree, *p*<0.001
Employee (permanent)	18 (15%)	27 (23%)	73 (62%)	
Employee (relief)	8 (21%)	16 (41%)	15 (38%)	
Total	31 (14%)	80 (35%)	117 (51%)	
Inspection fit for purpose				
Owner of RPB	18 (25%)	36 (51%)	16 (23%)	Owners of RPBs more likely to disagree, *p*<0.05 Employee pharmacists (permanent) more likely to agree, *p*<0.05
Employee (permanent)	59 (50%)	46 (39%)	13 (11%)	
Employee (relief)	13 (33%)	19 (49%)	7 (18%)	
Total	90 (39%)	101 (44%)	36 (16%)	
Appropriate to have unannounced inspections				
Owner of RPB	12 (17%)	57 (80%)	2 (3%)	Owners of RPBs disagreed more, statistical significance not demonstrated, NR
Employee (permanent)	49 (42%)	63 (53%)	6 (5%)	
Employee (relief)	11 (28%)	26 (67%)	2 (5%)	
Total	72 (32%)	146 (64%)	10 (4%)	
Effectiveness of PAS*				
Owner of RPB	48 (41%)	53 (45%)	17 (14%)	Employees (relief) more likely to agree, *p*<0.05
Employee (permanent)	28 (39%)	35 (49%)	8 (11%)	
Employee (relief)	17 (44%)	15 (38%)	6 (15%)	
Total	93 (41%)	103 (45%)	31 (14%)	
Effectiveness of new MPharm placement structure				
Owner of RPB	25 (35%)	26 (37%)	20 (28%)	NS, *p* = 0.881*
Employee (permanent)	56 (47%)	30 (25%)	32 (27%)	
Employee (relief)	16 (41%)	14 (36%)	9 (23%)	
Total	97 (43%)	70 (31%)	61 (27%)	
CPD—effectiveness of E-portfolio				
Owner of RPB	27 (38%)	36 (51%)	8 (11%)	Owners of RPBs more likely to have negative attitudes, *p* < 0.05
Employee (permanent)	71 (60%)	37 (31%)	10 (8%)	
Employee (relief)	17 (44%)	13 (33%)	8 (21%)	
Total	115 (50%)	86 (38%)	26 (11%)	
CPD—effectiveness of practice review				
Owner of RPB	19 (27%)	29 (41%)	23 (32%)	Employee pharmacists (relief) more likely to agree, *p*<0.05
Employee (permanent)	44 (37%)	51 (43%)	23 (19%)	
Employee (relief)	19 (49%)	9 (23%)	10 (26%)	
Total	82 (36%)	89 (39%)	56 (25%)	

*NR*: no result—Chi-squared test result unreliable

^#^*RPB* retail pharmacy business, *PAS Pharmacy Assessment System

*Not statistically significant (NS) at 5% significance level

Note: rounded percentages may not add to 100%, exact values do

#### Inspection/enforcement

Although the difference is small, more respondents (44%) considered that the current system of pharmacy inspection was not fit for purpose and did not protect the public interest, compared to those (39%) who did. RPB owners and those who had practiced under the preceding Acts were more likely to consider it ineffective than non-managerial staff (*p* < *0.05.* Two-thirds of respondents (64%) felt that “unannounced” inspections were unnecessary to adequately protect the public interest with those who practiced under the preceding Acts and RPB owners more likely to hold this view. Respondents were split in their view of the PSI’s PAS being an effective self-audit tool to support them delivering safe and effective care to their patients, with 45% disagreeing compared to 41% who felt otherwise.

#### Education and training

Close to half of the respondents considered that having two distinct practical training periods in the MPharm program positively enhanced preparation for independent community pharmacy practice. Regarding CPD, respondents mainly agreed that the ePortfolio was an effective method of ensuring that CPs remained competent to practice. Employee pharmacists (permanent) were more likely to consider this than owners or relief pharmacists (*p* < *0.05)* (Table 2). Respondents were divided as to whether the requirement for practicing CPs to undergo a practice review was effective in ensuring that professional competency was maintained, with more respondents considering it not effective (39% vs 36%).

### (b) Personal experience of regulation and the PSI

The majority of CPs (75%, *n* = 171) indicated that while the PSI’s community pharmacy practice standards accorded with what they considered was required to deliver a safe and effective service, it affected their workload. Most respondents (76%) indicated that the regulatory requirements hindered them from providing optimal care, either occasionally or frequently. Almost all respondents indicated that the attendant administrative tasks resulted in an increased workload (93%, *n* = 212), with 60% (*n* = 136) reporting a significant increase. 66% of respondents estimated this accounted for 3–7 h per week (*n* = 151), while 11% (*n* = 25) reported it at more than 10 h per week.

Regarding the level of overall trust CPs perceived the PSI has in them to discharge their professional activities competently and safely, some 45% (*n* = 102) perceived that it was a low level of trust and a medium level of trust for a further 41% (*n* = 93). RPB owners were more likely to report a low level trust than non-managerial pharmacists (*p* < *0.05*)*.* A third of respondents described their engagement with the PSI as being ‘fearful’ (34%), while a further 21% described it as “obstructive”.

Almost three-quarters of respondents considered that the current regulatory requirements acted as a disincentive to both recently registered pharmacists choosing to practice in community pharmacy (71%, *n* = 162) and to established CPs remaining in community practice (73%, *n* = 167). RPBs owners were more likely to consider the requirements a disincentive compared to employee pharmacists (*p* < *0.05*)*.*

A summary of the qualitative content analysis of the narrative responses obtained relating to the lived experience of the Pharmacy Act and the modified framework analysis based on the seven principles of better regulation is provided in Tables 3 and 4. In the main, corresponding themes in the form of both higher order categories and related categories identified in the original qualitative study also emerged in this study. However, a number of additional themes emerged from the analysis of the limited optional respondents’ comments relating to their “lived experience” of the Pharmacy Act and its implementation including overregulation; detrimental to patient care; perception of pursuit of minor matters; decision-making capacity of recent graduates and working conditions for CPs. The findings of this qualitative content thematic analysis together with illustrative respondent quotes are presented in Table 3.

**Table 3:** Higher order and related categories identified by qualitative content analysis—lived experience

Higher order category/theme	Related categories/themes	Illustrative quotes
Disciplinary provisions	PSI approach	“We are presumed guilty until proved otherwise….without regard for the years of good a pharmacist has given” “very punitive, creating an atmosphere of fear”
	Mediation	“I feel that the lack of use of mediation is a huge problem”
	Time delays	“I’ve been through a fitness to practice process…..The process dragged on for 5 years and hearings for 4 months”
	Pursuit of minor matters	“I sat for a number of years on the Conduct Committee and far too many trivial cases were brought forward that would have been easily dealt with via mediation…..”
Inspection/enforcement	Inspection approach/inspectors	“I feel they look at us as the bad guys they have to protect the public against.’’ “Inspectors seem to keep looking until they find something, they are not happy with”
	Pharmacy Assessment System Effectiveness/repetitive	“I don't know of a single pharmacist who doesn't just copy and paste their previous efforts. This is just an administrative waste of time….’’ ’It was good the first, and, perhaps, the second time…but it loses its benefits after a few repetitions.. needs to evolve…’’
	Diverts from patient care	“I find it a form filling exercise and something that interferes with providing a service to our customers…’’
Training and education	Integrated MPharm Programme	
	4- and 8-month blocks of practical training	“…anybody who hasn't had a full year of exposure with increasing responsibility during it, will find it difficult to assume full responsibility for pharmacy on registration” “new system…rushes the learning process”
	Wider experience of practice	“The new system gives students a broader experience of practice compared to the older 12-month…system “
	Decision-making skills of recently qualified pharmacists	“…they’re afraid to practice. They’ve been browbeaten during their training into upholding the “rules” at all costs…are unwilling or even afraid to actually think or make a judgement call”
	CPD	
	Effectiveness	“Only positive change I have seen in 30 years.…” ‘’The ePortfolio doesn't actually reflect what learning is done by pharmacists…” ’I doubt the ability for it [Practice Review] to actually highlight pharmacists who lack competence and it's an over intrusive, anxiety-inducing requirement for those who are competent already’’
	Convoluted	“I find the ePortfolio confounding. It’s too hard to use. I shudder to think of older pharmacists’ experience’’ “time-consuming” “overly onerous record-keeping involved”
	Practice review	“Someone may be competent in day-to-day practice but have a difficult experience at the practice review given that it is exam conditions…not sure how fair a process it is to be re-examining people who have a professional qualification” “Waste of time and money for all involved…absolutely ridiculous- would any other medical professional be asked to do the same and all at their own expense? NO!! ‘’…should only be used in cases where there is reason to suspect that a Pharmacist is not "up to the job"
Personal experience of regulation and regulator	Disconnect	“The requirement to be 100% compliant can occasionally give pause to the decision-making process…. The Pharmacy Act needs to acknowledge that pharmacists should be given a certain amount of autonomy” “Standards in the act….direct pharmacists to operate in a health system that is black and white…..healthcare doesn’t operate like that and the opportunity to use our professional judgment and experience isn’t appropriately considered”
	Administrative burden/detrimental to patient care	“Too much mindless paperwork is keeping me from practicing real patient-led pharmaceutical care”
	Overregulation	“Whilst I’m a firm believer in the regulation of my profession and understand the regulator’s societal duty of care, I feel there’s a clear and present danger of the overregulation of community practice” “Overregulation is now utterly endangering the safety of dispensing”
	Career disincentive	“….I am aware of at least one colleague who retired before he’d intended as a direct result of the manner by which he was treated during a CPD audit” “I absolutely hate being a CP because of the regulations” “The pressure and strain the PSI put on pharmacists is immense…Any young pharmacist should leave the profession if they have any sense coz [because] of the appalling way in which the PSI treats pharmacists”
	Fear	“I find young pharmacists are fearful and lack the confidence to make real-time professional interventions on patients’ behalves due to the regulations and their enforcement” “Many of our professional decisions are now made under a culture of fear of serious PSI sanctions rather than always in the best interest of the patient” “culture of fear and intimidation”
	Pharmacist working conditions	“Pharmacist working conditions needs to be better regulated…..it's not safe to allow pharmacists to work 11 h shifts with only a 5 or 10 min break all day. Excessive workload is one of the biggest risks to patient safety and it doesn't seem to be regulated by the PSI at all’’

**Table 4:** Principles of better regulation framework analysis—illustrative quotes

Principle of better regulation	Related categories/themes	Illustrative quotes
a. Necessity	Implementation	“The Act was necessary…Its interpretation is excessive & costly.’’
b. Effectiveness/targeted	Targeted approach to enforcement	“Approach to enforcing the act has been to publish and report on all negative aspects of pharmacy and pharmacists…..pharmacists are constantly trying to “watch their backs”” “Currently enforcement takes a "gotcha" approach, looking for the undotted i or uncrossed t, instead of focusing on…..delivering safe and effective care to patients’’
c. Proportionality	Enforcement proportional to risk posed to public health	“There is little evidence of a serious risk to public health from the actions of community pharmacies…Yet the Act is enforced as if community pharmacy poses a serious danger to those who use it”
	Assessment of risk	‘’I am not aware of any such published risk assessment carried out by PSI’’
d. Transparency	Stakeholder consultation	“I find the flow of consultations completed online are directed towards the ideal response as espoused by PSI—and it seems that they are only an exercise that needs to be done and that outcomes feel predetermined’’
e. Accountability	Accountable to whom and for what? Accountability of non-pharmacist owners of pharmacies	“Don’t answer to anyone….” “The PSI need to target and take on the Owners of Pharmacies where the owners are not Pharmacists. The Owners have the power to change whereas the Pharmacists don't, e.g., supply of resources’’
f. Consistency	Areas of inconsistency	“Chain pharmacies are given a light touch as they have bigger legal firms defending them” “I've seen a variance first hand with authorised officers depending on the pharmacy in particular rather than the legislation”
g. Agility	Scope of Practice	“The lack of increased scope of practice has caused many patients to be left without essential services, e.g., treating other minor ailments” “Colleagues in the UK laugh when I compare services we can and cannot supply in comparison to care that is 10 years in action elsewhere’’
	IT Advances	“Not keeping pace with desire from profession to innovate. Not keeping pace with technological advances—electronic prescribing”

### (c) Understanding of principles of better regulation

**Fig. 1:**
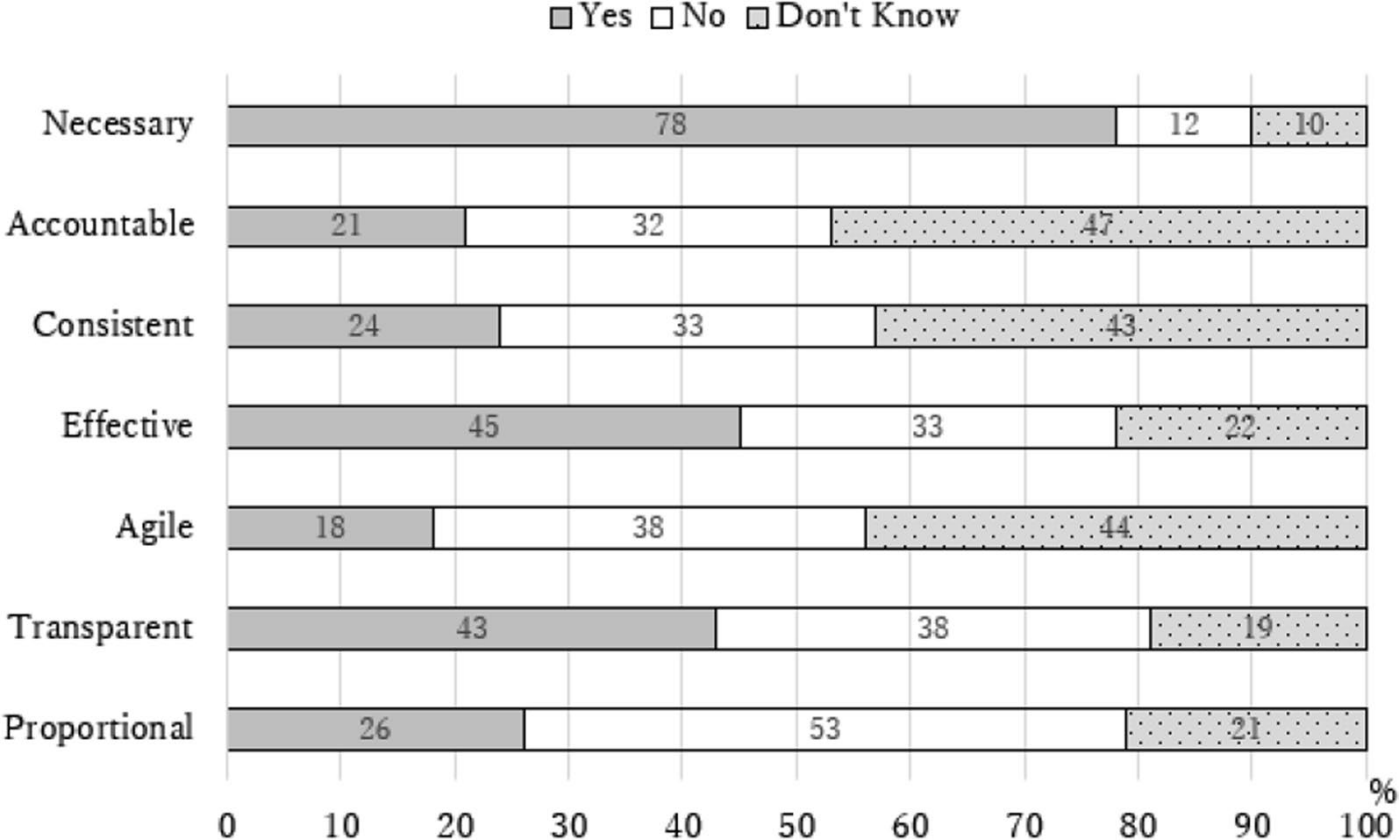
Respondents’ understanding of Better Regulation Principles related to the Implementation of the Pharmacy Act 2007

Respondents were presented with explanations of the seven principles of better regulation and asked whether they agreed or disagreed with the statement that the Pharmacy Act 2007 fulfills each of the principles (Fig. 1).

A substantial majority of respondents (78%, *n* = 176) considered that the Pharmacy Act was necessary, with no alternative means of protecting the public interest (78%, *n* = 177). Less than half of respondents (45%, *n* = 99) agreed that the Act was effective in regulating the profession and practice of pharmacy in the public interest, while 33% (*n* = 72) and 22% (*n* = 49) disagreed or were unsure, respectively. A greater proportion of respondents (53%, *n* = 115) did not feel that the PSI’s approach to enforcement of the Act was in proportion to the risk posed to public health by the delivery of community pharmacy services. Among this cohort of respondents, those who had practiced under the preceding Acts were more likely to disagree with this, (*p* < *0.05),* as were RPB owners (more so than employee CPs), (*p* < *0.05).* Similarly, a greater proportion of respondents (45%) felt that the PSI did not appropriately assess the risk posed to public health by the delivery of community pharmacy services.

Some 43% (*n* = 93) of respondents felt that the PSI implemented the provisions of the Act in a transparent manner, while 38% (*n* = 82) did not. RPB owners were more inclined to disagree than employee CPs (*p* < *0.001).* Most CPs (60%, *n* = 56) surveyed felt that the provisions of the Act, any associated secondary legislation and practice guidance were presented in a clear and accessible form to CPs. Regarding consultation processes conducted under the Act, more respondents felt that they were inadequate (43%, *n* = 98)) compared to those who considered they were adequate. Respondents who had practiced under the preceding Acts were more likely to disagree that the PSI act on feedback from consultations than those who had not, (*p* < *0.05)*, as were RPB owners, (*p* < *0.05).* Of the third of respondents who indicated that they had participated in a PSI consultation process 54% (*n* = 125) considered that their views had not been taken into account by the PSI when finalizing its position on the matter which it had consulted on.

A significant proportion of respondents, (47%, *n* = 107) did not know whether the PSI was appropriately accountable for its implementation of the Pharmacy Act, while a further 32% (*n* = 73) felt it was not. RPB owners were more likely to be in the latter cohort compared to employee pharmacists (*p* < *0.05).* The absence of awareness around accountability was reflected in the lack of knowledge around the availability of an appeals process with 55% (*n* = 125) indicating they did not know if one was available, while 21% (*n* = 49) believed that there was no appeals process available to CPs.

A lack of knowledge was again reflected in that the majority of respondents indicated that they did not know if the provisions of the Act were implemented by the PSI in a consistent manner across all RBPs (43%, *n* = 98). Some 33% (*n* = 76) did not think the approach was consistent while 24% (*n* = 54) considered it to be so. Over one-third of respondents did not consider that the Act, as implemented by the PSI, represented an agile model of regulation sufficiently capable of anticipating and adapting to change in CP practice.

A summary of the modified framework analysis of the limited optional free-text comments relating to the better regulation principles together with the related themes or categories that emerged with illustrative quotes is provided in Table 4.

## Discussion

There is a general absence in the literature of studies which examine the regulation of pharmacists, in particular from the perspective of professionals themselves, whose practice is directly impacted by such models of regulation and how they are implemented. Following on from earlier research by Lynch and Kodate (2020), this study provided all CPs practicing in Ireland with an opportunity to engage in the “*regulatory conversation*” and to report on their “*lived experiences*” of the Pharmacy Act regulating their practice [44]. Notwithstanding that many of them did not avail of the opportunity to participate as the authors would have wished (which is further considered below), the findings of this study provide an important insight into how a model of regulation, introduced to protect patients, can in the manner of its implementation be perceived by those it regulates as hindering their delivery of optimal care to the patients it seeks to protect.

A significant majority of those who responded agreed that the Act was necessary to regulate the profession and also considered that it was effective and transparent, which reflects the findings of Lynch and Kodate’s earlier study [44]. For the remaining principles of proportionality, accountability, consistency and agility however, most respondents indicated that the Pharmacy Act did not fulfill these. This, to a large extent, appears to be related not to the Act’s provisions *per se,* but rather to how they viewed its implementation by the PSI. The survey results also showed that respondent CPs’ understanding of the “better regulation” principles of agility, consistency and accountability may be difficult to identify with over 40% of respondents not knowing whether the Act fulfills these principles or not. However, their understanding of the principles of transparency, proportionality and effectiveness was higher. These findings illustrate differences between different types of pharmacists as seen for example, in that the majority of CPs who owned RPBs felt that the current model of regulation was inclined towards overregulation. This indicates that CPs are not a monolithic group, and their attitudes can vary depending on their experiences and employment status, which would merit further research.

Compared to other English-speaking countries regulation was underdeveloped in Ireland for many decades, particularly for the pharmacy profession. This delayed development could explain the tendency towards the perceived lack of proportionality in Ireland, as the regulator seeks to compensate for historical deficiencies in the regulatory framework. Respondents highlighted what they perceived as a culture of uncompromising “*black and white*” regulation within the sector. Many CPs appear to view the PSI as imposing various requirements to be strictly complied with in all circumstances, without affording CPs any professional discretion to act in what they considered was the best interests of their patient in a given situation. Such an approach runs contrary to the accepted characterization of a professional as someone who is able to apply discretion in their practice and use their judgement in the discharge of their professional activities [56, 57]. A high proportion of respondents reported their practice being hindered occasionally or frequently by the manner of the PSI’s implementation of the Act. CPs referred to the significant administrative burden associated with complying with the regulatory model and the deleterious effect of this on the service they provide to their patients, by diverting them away from routine face-to-face patient engagement. This is reflected in the reported hours of work spent on administration associated with complying with the Act and its provisions. The balance between rigidity (e.g., high level of standardization) on one hand, and flexible adaption and professional discretion on the other, in the current regulatory arrangement as determined by the PSI, appears tilted towards the former [27, 29]. Narrative responses to open-ended questions illuminated this by capturing the “lived experience” of CPs with the Pharmacy Act and its implementation by the PSI. These shed light on the meaning of social regulation in the health domain, as the delivery of care shifts more towards a person-centered, integrated and community-based model internationally. It also demonstrates the desirability of extending “regulatory conversations” as part of the process to include those who are regulated. This would promote a regulatory system in Ireland and elsewhere, that functions and regulates “better” and would serve as an incentive for positive behavioral changes among healthcare professionals [28, 58, 59].

In the original study, Lynch and Kodate concluded that the lived experience of CPs with the PSI’s inspection and disciplinary processes aligned more closely with that of a deterrence regulator than a compliance one [44]. Deterrence regulators view its regulatees as complying only when confronted with punitive sanctions, whereas compliance regulators use persuasion and support to encourage adherence to regulation [43, 60]. This study yielded similar findings with almost half of those who responded feeling that the disciplinary provisions of the Pharmacy Act were implemented by the PSI in a manner that did not appropriately balance protecting those in receipt of pharmacy services, with upholding the right of CPs to fair procedures. Many CPs perceived that the PSI pursued relatively minor cases through the full disciplinary process, and to adopt an adversarial approach to law enforcement. Several respondents commented on the protracted nature and delays often associated with these disciplinary processes which have been shown to increase the stress on those involved [61]. Importantly, many respondents believed that the PSI did not appropriately utilize the mediation provisions of the Act to resolve complaints, instead preferring to pursue the formal disciplinary route. Further study should examine the prevalence and characteristics of pharmacists who are subject to complaints, and also to compare the regulatory framework in Ireland with those in other English-speaking countries [17, 21].

The conduct of routine inspection of RPBs by the PSI, are predominantly undertaken without prior notice, i.e., unannounced. While close to half of respondents considered that the PSI’s current inspection system was effective in ensuring that the provision of CP services was fit for purpose and protects the public interest, more than two-thirds of CPs indicated that “unannounced” inspections in their view were unnecessary to adequately protect the public interest. CPs commented that inspections appeared to be conducted with the objective of finding deficiencies as opposed to supporting CPs to provide optimal pharmaceutical care.

Respondents were divided as to the effectiveness of the PAS introduced by the PSI in 2017 to complement and support its inspection process. The PSI viewed its introduction as it *“moving towards a more risk-based approach to inspection”.* For the period 2018 to 2020, as part of its commitment to developing *“outcomes-based, responsive, targeted and proportionate regulatory processes”,* the PSI has signaled its intention to further develop the PAS *“as a positive driver for quality improvement within pharmacy businesses”.* While this is ostensibly accurate, the PAS at present is primarily a system of self-audit against a list of requirements encompassing the sale and supply of medicines, documentation and RPB premises’ requirements. To be truly risk-based, the PSI would need to redesign it to focus more specifically on a range of proactive and reactive indicators of risk [62]. Such an inspection system based on a formal assessment of risk would identify those RPBs most in need of inspection and would support the PSI relaxing its requirement for all inspections to be unannounced.

In its Corporate Strategy 2018–2020, the PSI noted that *“we value, appreciate and respect everyone that we engage with”* and “*we work in partnership with our colleagues and all our stakeholders*” [63]*.* However, one-third of those surveyed selected “*fearful*” and just over a fifth chose “*obstructive*” when asked to describe the nature of their engagement/relationship with the PSI. When asked to assess the level of trust they considered the PSI had in them to discharge their professional activities competently and safely, CPs predominantly responded that they considered the PSI had either a low or medium level of trust to do so in the best interests of their patients, with only a small number indicating that they perceive the PSI has a high level of trust in them in this regard. As previously referred to, the incorporation of trust in the model of regulation is important. Lack of trust within the regulatory model tends to give rise to overregulation as the regulator seeks to compensate for its apparent lack of trust in the professional competence of those it regulates with yet further regulatory measures and requirements.

The OECD’s best practice principles for regulators recommends that regulators should be accountable to government or legislative oversight bodies and that there should be an appeals mechanism available for those regulated [39]. This study found that among those surveyed, many perceived the PSI as being largely unaccountable with no effective process available to them to appeal a finding of the PSI in any matter other than a decision to impose a sanction, an admonishment or a censure in a disciplinary hearing. In addition, the majority of CPs were unaware whether they could appeal a decision of the PSI that had a bearing on their practice. In their open comments, some respondents contrasted the apparent lack of accountability of non-pharmacists who beneficially own RPBs through company ownership, with the very tangible way in which they, as CPs, are held to account by the PSI for the operation of a RPB.

The PSI’s requirement in 2014 for a 5-year program of studies altered the previous practical training requirements. While a significant number of respondents considered this change enhanced the preparation for independent practice, quite a high proportion did not, with one CP noting that the 8-month training period in Year 5 “*rushes the learning process”*. The long-term impact of the MPharm program remains to be seen and needs continual review into the future.

The introduction of mandatory CPD was one of the more significant changes introduced by the Act and one which was generally viewed as positive by survey respondents. Regarding the model of CPD introduced by the PSI, respondents considered maintaining a reflective ePortfolio to be effective. However, a number of CPs referred to the complicated format associated with its completion, describing it as *“confounding”* while others felt it further added to the administrative burden associated with regulation referring to it as *“onerous”* and *“time-consuming”*. A recent study from the Netherlands highlights the association between pharmacist motivation and the features of their CPD program [64]. Respondents were less positive about the practice review, querying why pharmacists were the only healthcare professionals in Ireland required to undergo this form of review as part of their CPD. The practice review appears to introduce a de facto requirement for professional revalidation defined as *“the process by which assurance of continuing fitness to practice of registrants is provided and in a way which is aimed primarily at supporting and enhancing professional practice”* [65]. The PSI, in its Corporate Strategy 2021–2023, acknowledges that it is necessary to review its model of CPD *“to ensure that it supports future pharmacist practice in all its settings”* and that it is *“agile, adaptive and sustainable and that it delivers value for money”* [66]*.* The UK’s Professional Standards Authority advocates that regulators take a proportionate approach when developing suitable continuing fitness to practice mechanisms, based on a clear assessment of the level of risk of harm in the context of where the regulated group operates [48]. Such measures should be clearly targeted at areas of risk in performance. The current practice review is based on that originally used by the Ontario College of Pharmacists (OCP) which involves a knowledge assessment and an OSCE in a simulated setting [67]. Since 2019 however, the OSCE component in the OCP model has been replaced by a practice assessment in the pharmacist’s practice setting. Various CPs commented that the conduct of practice reviews should be targeted at those pharmacists whose standard of practice is giving rise to concern, rather than randomly selecting pharmacists following the review of their e-portfolios.

Almost three-quarters of those surveyed felt the regulatory requirements as implemented by the PSI acted as a disincentive either to recently registered pharmacists pursuing a career in community pharmacy or to established practitioners remaining in community practice. However, attrition from the pharmacy profession has been noted in other jurisdictions and attributable reasons are varied [68, 69]. In its Corporate Strategy 2021–2023, the PSI proposes to identify and mitigate risks to the continued availability of the professional community pharmacy workforce as an action under its strategic objectives**.** Aligned to this was a newly identified theme in this study not captured in Lynch and Kodate’s qualitative interviews. It relates to what respondents perceive as the apparent lack of regulation or concern of the PSI with the working conditions of pharmacists. They referred to long working hours without breaks and how this adversely affected their capacity to discharge their professional activities**.** These unfavorable working conditions may also contribute to pharmacists being less likely to choose, or continue to choose to practice as a community pharmacist. Arising from the findings of this study, the PSI might consider including a review of its approach to enforcing the Pharmacy Act to ensure that it does not act as a disincentive to pharmacists pursuing careers in community pharmacy.

The PSI “*commits to develop new ways to report to stakeholders on the learnings and data gathered through our regulatory work in pharmacies and inform the public as to how pharmacies are performing against PSI standards” *[66]. Engaging with stakeholders is an aspect of the better regulation principle of transparency, but many respondents in this study did not consider that the PSI takes adequate account of the views of stakeholders when it engages with them in consultative processes. Therefore, the PSI may need to reflect on its stakeholder engagement and transition from simply reporting or informing on findings, into something that is meaningful and tangibly reflects CPs’ contributions and concerns on the subject in question as highlighted in this study.

## Limitations and strengths

The authors acknowledge the very low response rate in this survey and the potential for this to impact on the generalizability of the findings to represent the wider CP population. However, the response rate is similar to a study of pharmacists on an analogous topic conducted recently in New Zealand [68]. The major contributing factor to this was undoubtedly the timing of the study in early 2020 which coincided with the onset of the COVID-19 pandemic when CPs were confronted with substantial challenges in maintaining essential services and public concerns. As frontline healthcare workers, they experienced profound changes to their daily practice to ensure continued provision of medicines and information to allay patient concerns as the pandemic developed, which is very likely to have detracted from their ability or inclination to engage with the study. A degree of response bias may have been introduced as the majority of our respondents were older, owners of RPBs, had practiced under the old Pharmacy Act or were at management grade level [70, 71]. These CPs may have been more likely to self-select to participate and provide their perceptions on the new Act and its implementation. However, many of these CPs have considerable experience in providing patient care in community pharmacy practice careers that in some instances predate the introduction of the Act in 2007, which it is considered would increase the likelihood of them wanting to engage with how the various regulatory changes and their implementation has impacted on the way they practice their profession and provide care to their patients. While the views and perceptions of these experienced CPs may be contested, they should not be dismissed and are valid insofar as the views as expressed make an important contribution to this nascent field of research and the developing literature in the area. The additional qualitative data (Table 3) collected in the form of free text proved useful, as these illustrate their viewpoints. In contrast, younger pharmacists at non-management grade, with less experience of the practical aspects of the implementation of the Act or not having practicing under a different regulatory model, may not yet be as inclined to consider how the Act and its implementation affects their ability to provide optimal patient care and accordingly may have self-selected not to complete the survey [70].

Despite these limitations, the responses from this larger study provide an enhanced insight into the perceptions and attitudes to the Pharmacy Act among CPs, further building on the findings from the previous in-depth qualitative study by Lynch and Kodate [44]. It is clear that the majority of CPs who responded acknowledge the necessity of the regulatory activities, although they question their proportionality and remain unsure or uninformed of the other aspects such as agility and accountability. Despite the potential bias of the study sample, the study represents the first nationwide questionnaire in this field either in Ireland or internationally and identifies key matters that warrant reflection and consideration not only by the PSI, but more widely by all healthcare regulators. In its Corporate Strategy 2021–2023, the PSI has committed to achieving reform of the Pharmacy Act and has signaled its intention to “*reviewing models of pharmacy regulation and other relevant healthcare systems” *[66]. The findings of this study and the earlier one by Lynch and Kodate provide an important contribution to supporting and informing the PSI’s proposals in this regard. Exploring specifically the perceptions and knowledge among younger members of the profession to regulation and its implementation, and further eliciting reasons for the perceptions provided among older and more experienced pharmacists is merited. A comparative and international collaborative study to gain insights into perceptions in geographically diverse sectors would be useful as it is acknowledged that there is a dearth of robust research in this area [17, 72].

## Conclusion

While health professional regulation must principally be concerned with public protection, it must also have regard for the impact of its requirements on those same professionals delivering frontline health services to patients. While CP respondents in this study unequivocally endorse the necessity of regulation as provided in the Pharmacy Act, their day-to-day experiences of its implementation suggest that a more responsive form of regulation is needed. A regulatory conversation where their views and concerns would be taken into account would serve to achieve this. As the PSI seeks to deliver on its strategic aim of evolving to a more effective regulatory model, this may be supported by it adhering more closely to the principles of better regulation, and by engaging in a regulatory conversation to identify and address perceived concerns about the model of regulation including overregulation; lack of risk-based assessment; failure to acknowledge competency and disproportionate and punitive disciplinary responses. However, these findings have resonance for health professional regulatory authorities in all countries that wish to discharge their regulatory functions in a responsive manner and seek to achieve the optimal balance in their regulatory approach between rigidity and flexibility. A regulatory conversation should enhance responsiveness by infusing a more dynamic mechanism for balancing the two (i.e., rigidity and flexibility). This study addresses some of the methodological challenges of conducting research in this area by providing an effective framework with which to conduct future research on models of healthcare professions that pertain internationally. This in turn can inform their implementation, future change and policy development, particularly with regard to fostering trust and accountability in the sector.

## Data Availability

The datasets generated and/or analyzed during the current study are not publicly available due to the fact that participants were specifically informed that their responses would remain confidential but are available from the corresponding author.
